# You’re Prettier When You Smile: Construction and Validation of a Questionnaire to Assess Microaggressions Against Women in the Workplace

**DOI:** 10.3389/fpsyg.2022.809862

**Published:** 2022-03-16

**Authors:** Mona Algner, Timo Lorenz

**Affiliations:** Department of Psychology, Medical School Berlin, Berlin, Germany

**Keywords:** scale development, genetic algorithm, test validation, sexism, diversity, gender microaggressions, women at work, confirmatory factor analyses

## Abstract

Gender microaggressions, especially its subtler forms microinsults and microinvalidations are by definition hard to discern. We aim to construct and validate a scale reflecting two facets of the microaggression taxonomy: microinsults and microinvalidations toward women in the workplace, the MIMI-16. Two studies were conducted (N1 = 500, N2 = 612). Using a genetic algorithm, a 16-item scale was developed and consequently validated via confirmatory factor analyses (CFA) in three separate validation samples. Correlational analyses with organizational outcome measures were performed. The MIMI-16 exhibits good model fit in all validation samples (CFI = 0.936–0.960, TLI = 0.926–0.954, RMSEA = 0.046–0.062, SRMR = 0.042–0.049). Multigroup-CFA suggested strict measurement invariance between all validation samples. Correlations were as expected and indicate internal and external validity. Scholars on gender microaggressions have mostly used qualitative research. With the newly developed MIMI-16 we provide a reliable and valid quantitative instrument to measure gender microaggressions in the workplace.

## Introduction

Although since the 1960s and 1970s organizations and lawmakers alike have implemented policies to reduce gender discrimination, movements in which women speak up against sexual harassment and abuse in the workplace are on the rise (e.g., #MeToo, Time’s Up) indicating the continuing existence of sexism (Diehl et al., 2020). A recent study by the German Federal Ministry of Family Affairs, Senior Citizens, Women and Youth presented supporting evidence: 63% of women (compared to 49% of men) experienced or witnessed some form of sexism in their direct environment (Wippermann, 2019).

### Microaggressions

There is an argument that sexism has morphed into a more ambiguous form (Dovidio and Gaertner, 2000; Nguyen and Ryan, 2008; Sue, 2010a). Discrimination characterized by beliefs that women are inferior, sexist stereotypes and open acts of discrimination are becoming increasingly uncommon (Swim et al., 1995; Cortina, 2008). Hence, old-fashioned, blatant forms of prejudice, so-called *overt discrimination* are to be contrasted with more subtle forms of discrimination (Jones et al., 2016), referred to as *microaggressions*. Other related concepts include *incivility* (Lim and Cortina, 2005; Cortina, 2008), *subtle gender bias* (Tran et al., 2019) or *benevolent sexism* (e.g., flattering women while simultaneously implicitly emphasizing their inferiority; Dardenne et al., 2007). We will use the term *gender microaggressions* to account for gender discrimination from here on. Microaggressions have more recently been defined as “*brief and commonplace daily verbal*, *behavioral*, *and environmental indignities*, *whether intentional or unintentional*, *that communicate hostile*, *derogatory*, *or negative racial*, *gender*, *sexual-orientation*, *and religious slights and insults to the target person or group*” (Sue et al., 2007, p. 5). These actions are often unconscious and ambiguous in their intent to harm, making them difficult to pinpoint, yet they might be just as detrimental to the target as the more blatant forms of discrimination (Jones et al., 2016; Diehl et al., 2020). Microaggressions can be divided into three major categories: *microassaults*, *microinsults*, and *microinvalidations* (Sue et al., 2007; Sue and Capodilupo, 2008; Sue, 2010a).

#### Microassaults

Microassaults are conscious, explicit discriminatory actions (verbal, non-verbal, or environmental) with the intent to harm the recipient. They resemble so-called old-fashion racism or sexism, for example telling sexist jokes, referring to women as “bitches” (Sue, 2010b).

#### Microinsults

Microinsults are often unconscious communications or actions “*that convey stereotypes*, *rudeness*, *and insensitivity*” (Sue, 2010b, p. 31) demeaning a person’s gender identity. This includes mistaking female doctors for nurses (Sue and Capodilupo, 2008).

#### Microinvalidations

Microinvalidations describe communications that negate or exclude thoughts, feelings, or the experiential reality of a stigmatized person. Gender blindness or denying individual discrimination *via* statements like “*I am not sexist*, *I have a daughter*” fall into this category (Sue, 2010b).

### Gender Microaggressions

Gender microaggressions are defined as daily, commonplace indignities toward women (Nadal, 2010). Other concepts of sexism are objectification theory (Fredrickson and Roberts, 1997), benevolent sexism (Glick and Fiske, 1996, 2001) or everyday sexism (Swim et al., 2001). Research on subtle forms of sexism is not new (Nadal et al., 2013), still the concept of *gender microaggressions* can contribute to the existing literature in three ways. First, unlike previous studies (e.g., Swim et al., 2001), it integrates interpersonal, systemic and environmental discrimination into one framework considering a broad range of categories (Nadal, 2010; Sue, 2010b), mirroring the lived experience of women who encounter barriers on several levels (Diehl and Dzubinski, 2016; Fitzsimmons and Callan, 2016). Second, microaggressions can be conscious, unconscious, or even with good intent (Sue, 2010b). Third, the construct differentiates between levels of explicitness ranging from ambiguous microinvalidations to slightly more overt microinsults to explicit microassaults (Basford et al., 2013).

Since the 1980s women in the United States are obtaining more university degrees than men, yet only 18% of top leadership positions are held by women (Diehl and Dzubinski, 2016). In 2020, 27.8% of the board members and 7.4% of the CEOs of the largest publicly listed organizations in the European Union were female (European Institute for Gender Equality, 2020b). The same holds true for Germany, where approximately 51% of the graduates are female, while women represented only 14.7% of the board members of the 200 largest organizations in Germany, and 8.0% of the CEOs (Kirsch et al., 2022). In line with these numbers, a growing body of research suggests that despite efforts to foster equality (i.e., Equal Opportunities Act) gender microaggressions persist in the workplace (Jones et al., 2016; Tran et al., 2019).

One of the reasons for the continued gender inequity might be rooted in the fact that discrimination has morphed into subtler forms, which are more difficult to detect (Hebl et al., 2002) and hence are reported less (Jones et al., 2016). In a meta-analysis, Jones et al. (2016) found that subtle gender discrimination might be at least as detrimental as overt discrimination. Their results built on attributional ambiguity theory (e.g., Crocker and Major, 1989), which posits that members of a marginalized group find it difficult to discern whether harmful actions occur because of their marginal status or other unrelated reasons. Stigmatized individuals will attribute negative feedback to prejudice against their group in situations where the situation is clear rather than ambiguous, i.e., the negative experience associated with discrimination can more easily be externalized when discrimination is overt. In case of subtle gender microaggressions females might tend to internalize the experience (e.g., “it’s my fault”). According to their meta-analytic findings, Jones et al. (2016) report that experimental studies (e.g., Crocker et al., 1991; Barreto and Ellemers, 2005; Salvatore and Shelton, 2007; Tao et al., 2017) support the assumptions that subtler microaggressions might be even more stressful for the target resulting in negative effects on cognitive functioning, higher levels of anxiety, increase of negative mood and decrease of positive mood.

Subtle discriminatory behavior occurs more frequently than overt forms (Pearson et al., 2009; Yoo et al., 2010). It is their chronic nature that can make them more detrimental than their overt counterpart (Jones et al., 2016), which might be due to the accumulation of seemingly slight microaggressions resulting in serious impact for the target analogous to the concept of daily hassles (Cortina, 2008; King and Jones, 2016; Jones et al., 2017). Gender microaggressions are often hardly visible, which makes them difficult to prove (Sue, 2010b; Jones et al., 2016) and because of their subtlety tend to get trivialized (Sue et al., 2007).

#### Gender Microaggression at the Workplace

Gender microaggressions at the workplace can have costly consequences for organizations and female leaders alike (Diehl et al., 2020). Gender microaggressions, or other forms of subtle gender discrimination, have been shown to negatively affect job satisfaction (Cortina et al., 2001; Chan et al., 2008), well-being (Lim and Cortina, 2005; Brondolo et al., 2008), self-esteem (Nadal, 2010; Oswald et al., 2019), engagement, organizational commitment, professional self-efficacy (Dardenne et al., 2007; Jones et al., 2016), subjective feelings of competence at the workplace (Glick and Fiske, 1996, 2001) and workplace performance (Chan et al., 2008; Jones et al., 2014). Others found a positive relation with turnover intention (Elvira and Cohen, 2001; King et al., 2010).

Gender microaggressions are considered to be one of the main barriers for women’s professional advancement (Diehl et al., 2020), by keeping women from meeting their vocational potential (Nadal and Haynes, 2012), as well as reaching leadership positions (Ely et al., 2011; Jones et al., 2016). For example, compared to men women are less frequently perceived as having what it takes to be a leader (Eagly and Carly, 2007; Hoyt, 2010; Hoyt and Murphy, 2016). Other scholars have found that women in power are rated as less effective (Lucas and Baxter, 2012; Hoyt and Burnette, 2013; Hoyt and Simon, 2016), receive lower performance ratings, fewer rewards (i.e., salary, bonuses and promotions; Joshi et al., 2015) and are less likely to be hired in male-dominated jobs than men (Koch et al., 2015). Further, work performance of women is more scrutinized (Kanter, 1977; Ryan and Haslam, 2007; Brescoll, 2016) and women are held to higher standards when it comes to promotions compared to their male colleagues (Lyness and Heilman, 2006; Inesi and Cable, 2015; Hoobler et al., 2018).

These findings emphasize the necessity of instruments to measure gender microaggressions at the workplace. Not only to detect their presence, but to foster a better understanding of the challenges women face at the workplace, as well as facilitating the development of interventions to decrease them.

#### Measuring Gender Microaggressions

Gender microaggressions, especially its subtler forms microinsults and microinvalidations are by definition hard to discern. In the past, some scholars developed instruments to measure subtle forms of discrimination. For example, Cortina et al. (2001) examined the quality of workplace social environments in general: their *Workplace Incivility Scale* (*WIS*) assesses subtle forms of workplace harassment such as gossiping, spreading rumors or ignoring others, but does not specifically focus on gender. Other scales that do focus on gender are intended for use in specific areas of the workplace, such as women in leadership positions (*Gender Bias Scale for Women Leaders*; Diehl et al., 2020), women in academia (*Perceived Subtle Gender Bias Index*, *PSGBI*; Tran et al., 2019) or focuses more on old-fashioned overt sexism (e.g., nude pictures, women are better suited for raising children than working; Leskinen and Cortina, 2014).

## Aim of This Study

To our knowledge, there is no questionnaire to assess microaggressions toward women in the workplace. Hence, we sought to construct and validate a scale reflecting two facets of the microaggression taxonomy: microinsults and microinvalidations. We decided to exclude microassaults from our scale for several reasons: Not only is the prevalence of overt sexism declining (European Institute for Gender Equality, 2020a), it is also increasingly socially proscribed (Wippermann, 2019). Furthermore, laws like the General Act of Equal Treatment in Germany or Directive 2006/54/EC implement principles of equal opportunities and equal treatment of men and women in German and EU labor law, respectively. We are not arguing that sexism does not exist anymore, we are arguing that societal and legal progress makes it easier to discern and report overt gender microaggressions compared to their subtler counterparts. In excluding the microassault facet, we further follow the recommendations of several scholars to adapt the microaggression concept in general. They have questioned the inclusion of microassaults, since they are per definition not subtle in nature and further bear the risk of trivializing overt acts of discrimination (Minikel-Lacocque, 2013; Wong et al., 2014; Garcia and Johnston-Guerrero, 2015; Lilienfeld, 2017). To differentiate more clearly between the overt and covert nature of discriminatory actions, Donovan et al. (2013) suggested to label microassaults as macroaggressions instead.

### Construction of a New Gender Microaggression Scale

In a seminal manuscript, Loevinger (1957) proposed a theory-driven approach to scale construction involving three aspects of construct validity: substantive validity, structural validity, external validity. Amongst others, Simms (2007), took this framework and developed a guideline for contemporary scale development, defining construct validity as its guiding principle for each of the three phases. The different foci of each phase are (i) construct conceptualization and generation of an initial item pool, (ii) item selection and construct validity, and (iii) assessment of convergent, discriminant and criterion-related validity (Simms, 2007).

Following these principles, we divided the scale construction in three stages, using a mixed-methods approach to develop the *Microinvalidation and Microinsult Scale-16* (*MIMI-16*). Stage one included a review of the relevant literature in order to develop a theory-driven conceptualization of constructs. In a pre-study we conducted semi-structured one-on-one interviews with 13 women to generate insight in their experiences with gender microaggressions. Since we aimed to develop a scale that can be used in different work settings, we specifically wanted to recruit a diverse sample of women regarding their age (21–61 years) and occupation (e.g., attorney, police officer, and teacher). Integrating theory and results from the interviews, we generated an initial item pool of 102 items reflecting the microaggression subfacets microinsults and microinvalidations (Sue et al., 2007). Following Lilienfeld (2017), we included a male individual as member of a majority group in the item creation process to minimize the risk of being predisposed to endorsing the concept. We presented the original items to a diverse group of individuals to make sure the items were comprehensible and to establish content validity. Consequently, we excluded several items, resulting in an item pool of 68 items.

In the second stage, we selected items and established construct validity. Study 1 consisted of a quantitative survey, including the original item pool of the MIMI-16, demographics and three validation measures. We used an automated item selection procedure to reduce the original item to the final scale and cross-validated our findings using a split-sample. We hypothesized a strong positive relation between our newly created measure and the *WIS* (Cortina et al., 2001) and the *PSGBI* (Tran et al., 2019), respectively. We decided to include these two instruments, because they are conceptually similar but still distinct enough: the *WIS* focuses on uncivil behavior in the workplace (i.e., no gender focus, but work related) and the *PSGBI* assesses subtle gender bias, but in a specific work environment (i.e., academia).

In stage three, in order to establish external validity, we ran bivariate correlational analyses with relevant work-related constructs. To test the external validity of the MIMI-16 we selected several important psychological constructs–meaning of work, job satisfaction, work engagement, occupational self-efficacy, and turnover intention. Furthermore, we investigate construct validity by means of a multiple regression analysis to test the impact of microaggression on turnover intentions, controlling for job satisfaction and other control variables. The specific hypotheses regarding the associations between the MIMI-16 and these constructs are discussed below.

#### Meaning of Work

Human beings search for meaning and often do so through work (Aguinis and Glavas, 2019), i.e., they want to experience their work as personally significant and worthwhile (Lysova et al., 2019). A growing body of research has established the association between meaning of work and some of the most important organizational outcomes, e.g., work motivation, stress, job satisfaction, career development and performance (for reviews, see e.g., Rosso et al., 2010; Lysova et al., 2019). Meaning of work is typically conceptualized as significance, broader purpose, and self-actualization (Martela and Pessi, 2018). Others have defined it as self-actualization, belongingness, and sense of achieving goals (Feser et al., 2019). Microaggressions are established to have a negative impact on subjective feelings of competence at the workplace (Glick and Fiske, 1996, 2001) and organizational commitment (Jones et al., 2016). Previous studies found that microaggressions keep women from realizing their full vocational potential (Nadal and Haynes, 2012), which is conceptualized as part of self-actualization (Martela and Pessi, 2018). We thus expect a moderate negative correlation between microaggressions and meaning of work.

#### Job Satisfaction

How individuals think about and relate to their work, and more specifically, the assessment of the favorability of a job (i.e., job satisfaction) is one of the most prolific research areas in work and organizational psychology (Judge et al., 2017). Job satisfaction has been associated with several relevant organizational outcome measures, such as increased performance (Judge et al., 2001; Harter et al., 2002), higher citizenship behavior (Judge et al., 2017), decreasing turnover intentions (Judge and Klinger, 2008) and less absenteeism (Scott and Taylor, 1985). Prior research on the relation between gender microaggressions and job satisfaction suggests that gender microaggressions lead to job dissatisfaction (Foley et al., 2005; King et al., 2010; Moors et al., 2014). In a meta-analysis Chan et al. (2008) further reported corrected correlations between sexual harassment and job satisfaction of ρ = −0.30. Consequently, we expect a moderate negative correlation between microaggressions and job satisfaction.

#### Work Engagement

Work engagement is defined as a “positive, fulfilling, work-related state of mind that is characterized by vigor, dedication and absorption” (Schaufeli et al., 2002). Among the antecedents of work engagement are the perception of emotionally, culturally, and physically safe environments and self-efficacy (for reviews see Wollard and Shuck, 2011; Kim et al., 2013), all likely to be compromised in individuals experiencing microaggressions. In several experimental studies, Dardenne et al. (2007) found that benevolent, but not hostile sexism reduced motivation and cognitive performance of women. We expect a small to moderate negative correlation between gender microaggression and work engagement.

#### Occupational Self-Efficacy

Occupational self-efficacy refers to the confidence a person feels regarding their ability to successfully fulfill the tasks involved in their job (Bandura, 1977; Rigotti et al., 2008). Previous studies suggested that gender microaggressions have a negative impact on self-esteem (Nadal, 2010; Oswald et al., 2019) and occupational self-efficacy (Dardenne et al., 2007; Jones et al., 2014). Furthermore, experimental evidence showed that gender microaggressions negatively influenced women’s self-efficacy and that self-efficacy mediates the relation between gender microaggressions and workplace performance (Jones et al., 2014). We expect a moderate negative correlation between gender microaggressions and occupational self-efficacy.

#### Turnover Intention

Turnover intention is a withdrawal behavior and that has been linked with underidentification with work (e.g., Bakker et al., 2004). It has been defined as the “conscious and deliberate willingness to leave the organization” (Bothma and Roodt, 2012 p. 5). Employee turnover is costly (Tracey and Hinkin, 2008; Boushey and Glynn, 2012), not only because of separation fees, but also due to hidden costs such as productivity loss or increased error rate of overburdened workers (O’Connell and Kung, 2007). Previous studies suggest that gender microaggressions increase employees’ intent to leave (Foley et al., 2005; Szymanski and Mikorski, 2016). Hence, we expect a moderate positive correlation between gender microaggressions and turnover intention.

#### Control Variables

Core self-evaluations (*CSE*; Judge et al., 1997) represent the fundamental appraisals individuals make about themselves, especially about their own worthiness and capabilities (Chang et al., 2012) and comprise the subfacets self-efficacy, self-esteem, emotional stability and locus of control. CSE are considered a stable personality trait and have been linked to job satisfaction (Judge et al., 1998; Judge and Bono, 2001). We further control for the gender composition of the workplace.

### Method: Study 1

#### Participants: Study 1

Study 1 consisted of 500 participants of which 497 self-identified as female and three as non-binary. The participants averaged 39.16 years (*SD* = 12.56) and were predominantly from a higher education background with 69% (*n* = 321) holding a university degree. Half of the participants (*n* = 256) were employed full-time, another 36.4% worked part-time. The remaining 12.4% (*n* = 62) of the sample were either apprentice, civil servant or self-employed. On average participants worked 33.91 h per week (*SD* = 9.71) and had 15.20 (*SD* = 13.30) years of working experience. Regarding their current work, the majority of participants (80.4%) stated occupation in the groups “health care, social affairs, and education” (*n* = 145), “company organization, accounting, law and administration” (*n* = 110), “humanities, social sciences and economic sciences, media, art, culture and design” (*n* = 74) and “commercial services, retail, sales and distribution, hotels and tourism” (*n* = 73). Every sector of the classification of occupation (Bundesagentur für Arbeit, 2011) was represented at least once. The study was conducted in German and participation was voluntary, hence no incentives were supplied. Participants were recruited *via* personal and professional networks as well as several online social media platforms.

#### Materials: Study 1

##### Demographics

Participants were asked to state their age, gender, highest level of completed education, employment status, weekly working hours, how long they have been working and sector of employment encoded with the classification of occupations 2010 (Bundesagentur für Arbeit, 2011). We further asked the participants to rate the size of their place of residence and their place of work (ranging from 1 = *rural* to 5 = *metropolitan*), their personal feminist attitude (ranging from 1 = *not at all* to 5 = *strong*), how much they agreed that gender equality already exists (1 = *completely disagree* to 5 = *completely agree*), as well as the approximate ratio of men and women in their workplace (ranging from 1 = *predominantly male* to 5 = *predominantly female*).

##### Incivility

Incivility was measured using the German version of the *WIS* (Jiménez et al., 2018). *Via* eight items participants were asked to rate the frequency of supervisor incivility and coworker incivility, respectively (e.g., “*Ignored me or did not respect my opinion*”). Participants answered on a Likert-scale ranging from 0 (*never*) to 6 (*daily*). Cronbach’s alpha (α) and McDonald’s omega (ω_*t*_) were α = 0.93 and ω_*t*_ = 0.95.

##### Perceived Subtle Gender Bias

Perceived subtle gender bias was measured using the PSGBI, a scale originally intended for use in academia (Tran et al., 2019). The German version of this scale was derived using a standard translation-back-translation procedure. We further adapted the scale to be used in universal workplace settings (e.g., “*female faculty members*” was replaced with “*females*”). The 21-item measure included four facets of perceived gender bias: Gender Inequality, Collegiality, Mentorship, and Institutional Support. Participants rated statements such as “*Some people are not comfortable being subordinate to a woman*” on a 6-point Likert-scale ranging from 1 (*disagree*) to 6 (*agree*). Cronbach’s alpha and McDonald’s omega were α = 0.91 and ω_*t*_ = 0.94.

##### Meaning of Work

Meaning of work was measured with a German meaning of work scale (*SiA*, for “meaning of work” in German; Feser et al., 2019). The SiA included three dimensions of meaning of work: self-realization, belongingness, and justification. Participants were asked to rate how much they agree with statements such as “*I am blossoming at work*.” Answer scales ranged from 1 (*I do not agree at all*) to 6 (*I fully agree*). Cronbach’s alpha (α) and McDonald’s omega (ω_*t*_) were 0.92 and 0.94, respectively.

##### Microinvalidations and Microinsults

The newly developed *MIMI-16* was used to measure microinvalidations and microinsults. On a scale from 1 (*I do not agree at all*) to 6 (*I fully agree*), participants rated 68 items such as “*It happens that male colleagues continue a meeting after the women have left the room*” or “*I have been sexualized in a professional context*” (for the final items in the *MIMI-16*, please refer to Table 1).

**TABLE 1:** Items of the MIMI-16.

Factor	Nr.	German wording	English wording
invalidations	1	Es kommt vor, dass männliche Kollegen ein Meeting fortsetzen, nachdem die Frauen den Raum verlassen haben	It happens that male colleagues continue a meeting after the women have left the room
	2	Ich habe das Gefühl, dass man mir weniger zutraut, weil ich eine Frau bin	I have the feeling that people expect less of me because I am a woman
	3	Frauen bekommen Komplimente für ihr Äußeres, Männer für ihre Arbeitsleistung	Women get compliments for their appearance, men for their work performance
	4	Andere nehmen an, dass sich Familiengründung negativ auf die Arbeitsleistung von Frauen auswirkt	Others assume that starting a family has a negative impact on women’s work performance
	5	Meine Durchsetzungskraft wird im beruflichen Kontext negativ bewertet	My assertiveness is viewed negatively in a professional context
	6	Man hat mir schon einmal zu verstehen gegeben, dass meine berufliche Leistung anders bewertet wird, als die von Männern	I have been made to feel that my professional performance is valued differently from that of men
	7	Ich habe das Gefühl ständig meine berufliche Qualifikation beweisen zu müssen	I have the feeling that I have to prove my professional qualifications all the time
	8	Vorschläge werden eher akzeptiert, wenn sie von einem Mann geäußert werden	Suggestions are more likely to be accepted if they are made by a man
insults	9	Manchmal bekomme ich Komplimente, die ich als unangebracht empfinde	Sometimes I receive compliments that I consider inappropriate
	10	Unter meinen Kolleg*innen werden manchmal anzügliche Witze gegenüber Frauen gemacht	Among my colleagues, sometimes suggestive jokes are made toward women
	11	Es ist schon vorgekommen, dass Kolleg*innen meine Kleidung kommentierten	It has happened that colleagues have commented on the way I was dressed
	12	Ich wurde in meinem Arbeitsumfeld schon nach meinem Menstruationszyklus gefragt	I have been asked about my menstrual cycle at my workplace
	13	Ich bin im beruflichen Kontext sexualisiert worden	I have been sexualized in a professional context
	14	Es kam schon vor, dass man mir an meinem Arbeitsplatz anzügliche Kosenamen gegeben hat	It has happened that I have been given suggestive pet names at my workplace
	15	Mein Verhalten wurde schon einmal aufgrund meines Geschlechts scherzhaft nachgeahmt	My behavior has been jokingly imitated because of my gender
	16	Ich habe das Gefühl, dass mein Aussehen mehr für meinen beruflichen Erfolg verantwortlich ist, als meine Qualifikation	I feel that my appearance is more responsible for my professional success than my qualifications

*MIMI-16, Microinvalidations and Microinsults Scale.*

#### Data Analysis

We used an automated item selection algorithm to develop the MIMI-16. Since algorithmic approaches are not yet common practice in organizational and social sciences, we give a brief overview [for an in-depth introduction to metaheuristics in general and genetic algorithms in particular, please refer to Gendreau and Potvin (2010) and Reeves (2010), respectively]. Scale development, i.e., selecting items to create a psychometrically sound scale, can be defined as a combinatorial problem (Kerber et al., 2022). Combinatorial problems, such as the *knapsack problem* (“*Choose a set of objects*, *each having a specific weight and monetary value*, *so that the value is maximized and the total weight does not exceed a predetermined limit*;” Schroeders et al., 2016, p. 4) refer to the process of finding a discrete and finite solution given a set of constraints (Hoos and Stützle, 2005). Although the concept is most prevalent in economics (e.g., the well-known *traveling salesman problem*), it has recently been applied to the item selection process in psychological scale construction (e.g. Schultze, 2017; Kerber et al., 2022). In this context the problem can be understood as selecting a set of items from an original item pool that fulfills certain predefined criteria (e.g., building a two-dimensional scale with good model fit).

Contemporary approaches solve these combinatorial problems using automatic optimization algorithms such as *Genetic Algorithms* (GA; Holland, 1975) based on natural evolution. Instead of selecting items based on their unique qualities, as classical approaches do, these so-called heuristic item selection algorithms aim to improve the psychometric properties of a set of items given a predefined set of constraints (Schultze, 2017). One important aspect is the approximate rather than deterministic nature of metaheuristics (Blum and Roli, 2003). Thus, they cannot be understood as approaches that guarantee finding the single-best solution (Yarkoni, 2010). Yet, approximate algorithms are often the only solution to obtain near-optimal solutions for complex combinatorial problems in an appropriate time, or at low computational cost (Dorigo and Stützle, 2010). In other words, meta-heuristics are particularly useful because the psychometric criteria can only be computed in combination with other items, with the aim to improve the quality of the scale as a whole (Olaru and Danner, 2021). Recent findings in scale development or adaptation suggest that algorithmic approaches perform at least as well as traditional approaches (Sandy et al., 2014) or even outperform them (Schroeders et al., 2016; Olaru and Danner, 2021).

##### Item Selection Procedure

In this study we used a genetic algorithm to select items from our original item pool to develop the final version of the MIMI-16. GAs aim to reduce a large set of variables by employing stochastic search methods based on evolutionary processes, i.e., the chance of a solution to survive and reproduce, its fitness, determines its quality (Galán et al., 2013). They are based on two processes, variation, and selection. While the first fosters diversity and novelty, the second rewards quality. The idea is to eventually generate an optimal or near-optimal solution (Galán et al., 2013). Applied to scale development, the procedure starts with *genes*, each representing different parameters or variables. Combining the genes to a string, the resulting *chromosome*, can be understood as a set of items or scale. The algorithm creates an initial population by randomly generating a predefined number (typically 100–200 individuals) of chromosomes from the original item pool, thereby ensuring variability (Yarkoni, 2010). Because the overall goal is to construct a scale with good psychometric properties (e.g., maximal reliability and validity while also exhibiting a good model fit of the measurement model), the next step requires the definition of a *fitness function* to evaluate the quality of a solution. In every generation the fittest *chromosomes* are extracted as a breeding ground for the next generation. To increase genetic diversity, mutation, i.e., spontaneous change of items in a scale, and recombination, i.e., exchange of items between two scales, are frequently employed. In a predefined number of iterations (i.e., 100+), usually define the fittest chromosome as the optimal solution.

We used a genetic algorithm implemented in the R package “stuart” version 0.9.1 (Schultze, 2020) with the aim to construct a two-dimensional scale. The original dataset was randomly split into a training (*n*_1_ = 250) and a test dataset (*n*_2_ = 250). The solutions were evaluated against an objective function consisting of a combination of the model fit criteria Root Mean Square Error of Approximation (RMSEA), Standardized Root Mean Square Residual (SRMR) and the Comparative Fit Index (CFI) as well as a composite reliability computed as McDonald’s ω. In the next step we validated our findings using k-fold cross-validation with the test dataset using the “*crossvalidate*” function of the R package “stuart” (Schultze, 2020).

##### Evaluation of Model Fit, Measurement Invariance, and External Validity

Model fit is evaluated using standard recommendations proposed by Hu and Bentler (1999). These comprise of χ^2^ significance testing as well as a combination of several fit indices, i.e., RMSEA < 0.05, SRMR < 0.07, CFI > 0.95. The confirmatory factor analysis (CFA) is run with the R package “lavaan” (Rosseel, 2012). Preliminary analyses revealed that microinvalidations, microinsults, and the total scale gender microaggressions was only slightly non-normally distributed (microinvalidations: skew = 0.37, kurtosis = −0.74; microinsults: skew = 0.96, kurtosis = 0.32 and total scale: skew = 0.58, kurtosis = −0.37). To account for non-normal distribution, we used a robust maximum likelihood estimator (MLR). Furthermore, the selected scale will be validated using k-fold cross-validation, in order to examine whether the solution holds in a test sample with regard to the four standard measurement invariance assumptions based on Meredith (1993).

To evaluate divergent and convergent validity of the MIMI-16, Pearson’s correlation coefficients were calculated with other relevant measures. Correlations were evaluated as follows: correlations >0.1–small, >0.3–moderate, and >0.5–strong. Because we used a forced-choice answer format, no data was missing.

#### Study 1: Results

##### Demographic Results

On average, participants lived in rather urban environments (*M* = 3.54, *SD* = 1.45). Similar applied to the place of work (*M* = 4.00, *SD* = 1.17). Participants had more female than male colleagues (*M* = 2.93, *SD* = 1.17), self-identified as rather feminist (*M* = 3.66, *SD* = 1.03) and on average rated the current state of gender equality at 2.24 (*SD* = 0.78).

##### Descriptives and Correlations

Table 2 presents descriptive statistics, McDonald’s ω, Cronbach’s α and the correlation matrix for the respective variables. The strong correlations (*r* = 0.68–0.70, *p* < 0.001) between the newly created MIMI-16 and the incivility scale and the PSGBI, respectively, indicate the MIMI-16 measures a similar, yet distinct concept. As hypothesized, the MIMI-16 correlated moderately negatively with the SIA.

**TABLE 2:** Descriptives and inter-correlations for study 1.

	*M*	*SD*	MIMI-16 total	Microinsults	Microinvalidations	SiA	PSGBI	Incivility	Feminism	Equality
MIMI-16 total	2.58	0.96	0.91 (0.89)							
Microinsults	2.34	1.00	0.89***							
Micro-invalidations	2.82	1.13	0.91***	0.62***						
SiA	4.37	1.00	−0.39***	−0.25***	−0.44***	0.94 (0.92)				
PSGBI	3.01	0.93	0.71***	0.48***	0.78***	−0.58***	0.94 (0.91)			
Incivility	1.55	1.11	0.68***	0.56***	0.65***	−0.55***	0.72***	0.95 (0.93)		
Feminism	3.66	1.03	0.24***	0.19***	0.24***	0.05	0.15**	0.05		
Equality	2.24	0.78	−0.34***	−0.25***	−0.35***	0.12*	−0.33***	−0.23***	−0.21***	
WE	2.93	1.17	−0.21***	−0.15***	−0.23***	0.16***	−0.22***	−0.06	0.02	−0.03

*McDonald’s omega (Cronbach’s alpha) is displayed in diagonals if applicable.*

*MIMI-16, Microinsult and Microinvalidation Scale; SiA, meaning of work scale; PSGBI, Perceived Subtle Gender Bias Index; WE, work environment.*

*<0.05; **<0.01; ***<0.001.

##### Model Fit and Latent Structure in the Construction Sample

**FIGURE 1:**
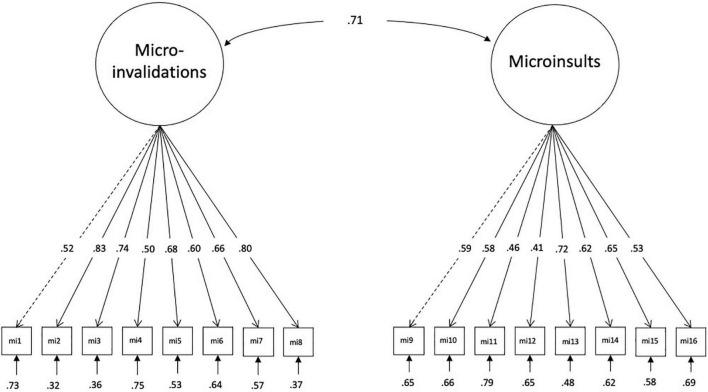
Measurement model for the MIMI-16 in the construction sample. MIMI-16, Microinsults and Microinvalidations Scale, abbreviated items refer to Table 1.

The GA selected 16 of the 68 original items representing the two factors microinvalidations and microinsults with eight items each (Figure 1). The final solution exhibits good model fit with Satorra-Bentler-χ^2^(103, *N* = 250) = 117.01, *p* = 0.163, CFI = 0.989, TLI = 0.987, SRMR = 0.043, RMSEA = 0.023, 90%-CI_*RMSEA*_ [0.000; 0.042]. Standardized loadings of the factor microinvalidations ranged from 0.50 to 0.83 and for microinsults from 0.41 to 0.72. All factor loadings including standard errors can be found in the Supplementary Material. Cross-validation with the second half of the data indicated that the assumption of strict measurement invariance holds across the two subsamples: χ^2^(252) = 366.34, *p* < 0.001, CFI = 0.960, SRMR = 0.056, RMSEA = 0.043, χ^2^ = 24.69, Δ*df* = 16, *p* = 0.076.

### Method Study 2

#### Participants: Study 2

Study 2 consisted of 612 participants of which 606 self-identified as female and six as non-binary with an average age of 37.16 years (*SD* = 9.45). In this study 72% (*n* = 441) hold a university degree, 49.2% of the participants (*n* = 301) were employed full-time and 35.9% worked part-time (*n* = 220). The remaining 14.8% (*n* = 91) of the sample were either apprentice, civil servant or self-employed. We excluded two values due to implausible answers regarding their weekly work hours. On average participants worked 34.58 h per week (*SD* = 9.86) and had 11.70 (*SD* = 10.09) years of working experience. The majority of participants (83.3%) stated their current occupation in the groups “health care, social affairs, and education” (*n* = 216), “humanities, social sciences and economic sciences, media, art, culture and design” (*n* = 130), “company organization, accounting, law and administration” (*n* = 88), and “commercial services, retail, sales and distribution, hotels and tourism” (*n* = 76). In this study the military sector was not represented. The study was conducted in German and participation was voluntary, hence no incentives were supplied. Participants were recruited on several online social media platforms.

#### Materials: Study 2

##### Demographics

Participants were asked the same demographic questions as in study 1.

##### Job Satisfaction

We measured job satisfaction with three items (Judge and Klinger, 2008). The first item assesses global job satisfaction with a dichotomous answer format (“*All things considered*, *are you satisfied with your present job?*”). The second item (“*How satisfied are you with your job in general?*”) measures the extent of satisfaction with the present job on a five-point Likert-scale ranging from 1 = *very dissatisfied* to 5 = *very satisfied.* With the third item, participants are asked to estimate the percentage of time they feel satisfied, dissatisfied, and neutral about their present job on average (“*The percent of time I feel satisfied with my present job*”). Job satisfaction was assessed with the mean score of the *z*-standardized items. Cronbach’s alpha (α) and McDonald’s omega (ω_*t*_) were 0.81 and 0.82, respectively.

##### Core Self-Evaluation

We measured *core self-evaluations* with the German version of the *Core Self-Evaluation Scale* (*G*-CSES; Heilmann and Jonas, 2010). The G-CSES consists of 12 statements (“*I am confident I get the success I deserve in my life*”). Participants rated these items on a five-point Likert-scale from 1 = *strongly disagree* to 5 = *strongly agree*. Cronbach’s alpha and McDonald’s omega were α = 0.84 and ω_*t*_ = 0.87.

##### Turnover Intention

Intention to leave their current job was measured with the *German Turnover Intention Scale* proposed by Böhm (2008). On a five-point Likert-scale ranging from 1 = *strongly disagree* to 5 = *strongly agree* participants rate three statements such as “*I often think about leaving my job at my current company.*” Cronbach’s alpha and McDonald’s omega were α = 0.86 and ω_*t*_ = 0.86.

##### Work Engagement

We used the *German Utrecht Work Engagement Scale-9* (UWES-9; Sautier et al., 2015) to measure work engagement. The UWES-9 consists of nine items (e.g., “*At my work*, *I feel bursting with energy.*”), which participants rated on a 7-point Likert-scale (from 0 = *never* to 6 = *always*). Cronbach’s alpha (α) and McDonald’s omega (ωt) were 0.93 and 0.95, respectively.

##### Occupational Self-Efficacy

Occupational self-efficacy was evaluated with the short version of the German Occupational Self-Efficacy Scale (OSS-SF; Rigotti et al., 2008). Six items, such as “*When I am confronted with a problem in my job*, *I can usually find several solutions.*” are rated on a six-point Likert-scale (from 1 = not *at all tru*e to 6 = *completely true*). Cronbach’s alpha (α) and McDonald’s omega (ω_*t*_) were 0.87 and 0.91, respectively.

#### Data Analysis

##### Evaluation of Model Fit, Measurement Invariance, and External Validity

The original dataset was randomly split into two sub-datasets (*n*_1_ = 306, *n*_2_ = 306). Model fit was evaluated by means of CFA using the same criteria as presented in study 1. We tested the four standard measurement invariance assumptions between the two datasets using the R package “psych” (Revelle, 2020). To evaluate divergent and convergent validity of the MIMI-16, Pearson’s correlation coefficients were calculated with other relevant measures. Correlations were evaluated as follows: correlations >0.1–small, >0.3–moderate, and >0.5–strong. Because we used a forced-choice answer format, no data was missing.

##### Regression Analysis

The data was checked for the necessary prerequisites to conduct multiple regression analysis. We used the R package “car” to assess the variance inflation factor (VIF). The VIF over all variables was good with scores between 1.08 and 1.28 (O’brien, 2007).

#### Study 2: Results

##### Demographics

Participants lived in rather urban environments (*M* = 3.77, *SD* = 1.18) and similarly applied to the place of work (*M* = 3.58, *SD* = 1.35). On average, participants had more female than male colleagues (*M* = 3.01, *SD* = 1.18), self-identified as feminist (*M* = 4.16, *SD* = 0.84) and rated the current state of gender equality in society at 1.87 (*SD* = 0.86).

##### Descriptives and Correlations

Descriptive statistics, McDonald’s ω, Cronbach’s α and bivariate correlations are presented in Table 3. As expected, the MIMI-16 exhibited a moderate negative correlation with core self-evaluations and job satisfaction (*r* = −0.32 and −0.32), as well as a moderate positive correlation with turnover intention (*r* = 0.31, all at *p* < 0.001). We expected a small to moderate correlation between the MIMI-16 and work engagement. The hypothesis was confirmed albeit smaller than expected (*r* = −0.15, *p* < 0.001). The negative correlation between MIMI-16 and occupational self-efficacy was *r* = −0.18, *p* < 0.001 and thus smaller than hypothesized.

**TABLE 3:** Descriptives and inter-correlations for study 2.

	*M*	*SD*	MIMI-16 total	Micro-insults	Micro-invalidations	UWES-9	OSS-SF	TIS	G-CSES	JS	Fem	Equal
MIMI-16 total	3.17	1.12	0.91 (0.89)									
Microinsults	2.97	1.18	0.92***									
Microinvalidations	3.38	1.24	0.93***	0.70***								
UWES-9	4.16	1.18	−0.15***	−0.15***	−0.13**	0.95 (0.93)						
OSS-SF	4.39	0.92	−0.18***	−0.15***	−0.18***	−0.51***	0.91 (0.87)					
TIS	3.04	1.29	0.31***	0.26***	0.31***	−0.48***	−0.26***	0.86 (0.86)				
G-CSES	3.42	0.63	−0.32***	−0.29***	−0.30***	0.42***	0.67***	−0.028***	0.87 (0.84)			
JS^a^	0.00	0.85	−0.35***	−0.30***	−0.34***	0.63***	0.39***	−0.65***	0.40***	0.82 (0.81)		
Fem	4.16	0.84	0.22***	0.18***	0.23***	−0.05	−0.07	0.10	−0.12**	−0.06		
Equal	1.87	0.86	−0.35***	−0.29***	−0.35***	0.14***	0.20***	−0.15***	0.25***	0.13**	−0.28***	
WE	3.01	1.18	−0.28	−0.23	−0.28	0.01	0.01	−0.05	0.02	0.08*	−0.05	−0.06

*McDonald’s omega (Cronbach’s alpha) is displayed in diagonals if applicable.*

*MIMI-16, Microinsult and Microinvalidation Scale; UWES-9, Utrecht Work Engagement Scale-9; OSS-SF, Occupational Self-Efficacy Scale-Short Form; TIS, turnover intention scale; G-CSES, German Core Self-Evaluation Scale; JS, job satisfaction; Fem, feminism; Equal, subjective equality; WE, work environment.*

^a^Standardized z-scores *<0.05; **<0.01; ***<0.001.

##### Model Fit and Latent Structure in Two Separate Validation Samples

Model fit of the newly developed MIMI-16 was good in both validation samples (numbers in squared brackets refer to fit indices in sub-dataset 2): CFI = 0.936 [0.960], SRMR = 0.049 [0.042], 90%-CI_*RMSEA*_ = 0.050–0.074 [0.038–0.064]. Measurement models for the MIMI-16 in all datasets are presented in Table 4. Standardized loadings of the factor microinvalidations ranged from 0.49 to 0.81 (sub-dataset 2: range [0.55;0.86]) and for microinsults from 0.44 to 0.78 (sub-dataset 2: range [0.43;0.76]). All factor loadings including standard errors can be found in the Supplementary Material, Table 2. Strict measurement invariance holds between the two samples [χ^2^(250) = 488.74, Δχ^2^ = 17.42, Δ*df* = 16, *p* = 0.359].

**TABLE 4:** Measurement models for MIMI-16 using MLR estimator^a^.

MIMI-16	*N*	χ^2^	*df*	*p*	CFI	TLI	SRMR	RMSEA	90%CI RMSEA
**Study 1**									
Dataset 2	250	150.34	103	0.002	0.959	0.953	0.049	0.046	0.029–0.062
**Study 2**									
Dataset 1	306	207.43	103	<0.001	0.936	0.926	0.049	0.062	0.050–0.074
Dataset 2	306	174.84	103	<0.001	0.960	0.954	0.042	0.051	0.038–0.064

*MIMI-16, Microinvalidations and Microinsults Scale.*

*^a^CFA calculated with Satorra-Bentler adjusted χ^2^.*

##### Regression Analysis

The model composed of job satisfaction, microaggressions, the work-environment, and core self-evaluation as predictors of turnover intention and was tested using multiple regression analysis (R_*adj*_ = 0.42). The results are in favor of our hypothesis. Job satisfaction (β = −0.61; *p* ≤ 0.01) and microagressions (β = 0.11; *p* = 0.02) are statistically significant predictors of turnover intentions while the work environment (β = 0.02; *p* = 0.38) and core self-evaluation (β ≤ 0.01; *p* = 0.90) do not become statistically significant predictors.

## Discussion

In this study, we developed and validated an instrument to assess microinsults and microinvalidations against women in the workplace using an automated item selection algorithm. In four distinct samples (*N* = 1,112) the MIMI-16 exhibited good psychometric properties. Furthermore, microaggressions were a statistically significant predictor for turnover intentions, even when it was controlled for job satisfaction, work environment and core self-evaluation.

### Factorial Structure

Following the recommendations of scholars in the past (i.e., Lilienfeld, 2017), by excluding the factor microassaults we reduced the complexity and adapted the existing conceptualization of the microaggression taxonomy. We developed a scale using a genetic algorithm with the goal to assess the two facets microinsults and microinvalidations. The *microinvalidations* factor consists of items focusing on the unequal standards women are held against compared to their male colleagues (e.g., women might have to prove themselves more and find their work overly scrutinized compared to men, Ryan and Haslam, 2007; Brescoll, 2016; Hoobler et al., 2018). The factor *microinsults* includes items that convey hostility such as sexualization, being made fun of or mentioning the menstrual cycle.

Another aspect that has been criticized before is the lack of factorial analyses in previous studies (Lilienfeld, 2017). We established factorial validity of the MIMI-16 by means of a CFA. The MIMI-16 exhibited good model fit in the construction sample, as well as in three validation samples. Multigroup CFA suggested that assumptions of strict measurement invariance hold between all samples. With this scale, we provide a valid instrument to empirically assess microinvalidations and microinsults against women.

### Correlations With Organizational Outcomes

We ran correlational analyses with several organizational outcome measures such as job satisfaction and turnover intention. The results correspond with previous studies (e.g., Chan et al., 2008; King et al., 2010). The MIMI-16 correlated negatively with *meaning of work*, *work engagement*, *occupational self-efficacy*, and *job satisfactio*n and positively with *turnover intention*. The data suggest a low association between the MIMI-16 and work engagement and occupational self-efficacy, respectively. This might point to the fact that women in general feel the need to work harder in order to fulfill the higher standard and receive promotions (Brescoll, 2016; Hoobler et al., 2018), regardless of their experience of microinvalidations and -insults. Another possible explanation for this result could be rooted in the fact that the majority of participants held a university degree, indicating the possibility that they are operating on a high level of professionalism.

### Limitations

Before discussing specific results of the study, we discuss some limitations regarding generalizability. First, we recruited participants using personal and professional social networks resulting in a non-probability sample. Although this strategy increases response rates and allows recruiting individuals from diverse backgrounds, it raises concerns regarding generalizability.

Second, in both studies we relied on self-report data, which tend to get criticized as being inherently biased. On the other hand, Chan (2009) argues that self-report data is not that flawed after all. We, too, believe women to be the best source of information when it comes to their lived experiences. Still, future research might have a look into developing multi-source instruments to gain further insights into the matter. We measured all variables *via* self-report at the same time, which poses the risk of common method bias (Podsakoff et al., 2003). Then again, other scholars argue that this assumption distorts and oversimplifies the true issue, doubting that common methods inflate correlation to any significant degree (Wagner and Crampton, 1993; Spector, 2006).

Third, the PSGBI was not available in German and was translated-back-translated by us. This technique was criticized before (Geisinger, 1994), in future studies it would be helpful to follow guidelines for cross cultural research (e.g., Bartram et al., 2018).

Fourth, the questionnaire was developed and validated in Germany, hence when applying the MIMI-16 in different cultural settings, scholars in the future should keep in mind that the manifestations of gender microaggressions might differ.

### Implications and Future Directions

Our research advances understanding of gender microaggressions in several ways. To our knowledge we are the first to provide a validated instrument to measure microinvalidations and microinsults against women in the workplace with the claim to be applicable for women in all positions and industries.

The existing body of literature on gender microaggressions has shed light on an often-overlooked area of bias. We add to the research on gender microaggression theory by adapting the existing threefold taxonomy thus integrating some of the conceptual concerns raised by scholars in the past (e.g., regarding the microassault factor, Lilienfeld, 2017). To our knowledge, scholars on gender microaggressions have mostly used qualitative research (Capodilupo et al., 2010; Lau and Williams, 2010). With the newly developed MIMI-16 we provide a quantitative instrument to measure gender microaggressions. Possible future studies should evaluate the impact of gender microaggressions using longitudinal study designs. For example, our data suggests a statistically significant moderate negative correlation (*r* = −0.32) between core self-evaluation and gender microaggressions. It might be worthwhile to further investigate the longitudinal interaction of the manifestation and quality of core self-evaluation with gender microaggressions, in order to potentially establish a causal direction.

Other questions of interest could include the effect of microinvalidations and microinsults across different levels of professionalism and organizational hierarchy, as well as on women at early stages of their career. Furthermore, the possible measurement of microinvalidations and microinsults allows the evaluation of organizational interventions to reduce the phenomena in organizations.

## Data Availability Statement

The original contributions presented in the study are included in the article/Supplementary Material, further inquiries can be directed to the corresponding author.

## Ethics Statement

Ethical review and approval was not required for the study on human participants in accordance with the local legislation and institutional requirements. Written informed consent for participation was not required for this study in accordance with the national legislation and the institutional requirements.

## Author Contributions

Both authors listed have made a substantial, direct, and intellectual contribution to the work, and approved it for publication.

## Conflict of Interest

The authors declare that the research was conducted in the absence of any commercial or financial relationships that could be construed as a potential conflict of interest.

## Publisher’s Note

All claims expressed in this article are solely those of the authors and do not necessarily represent those of their affiliated organizations, or those of the publisher, the editors and the reviewers. Any product that may be evaluated in this article, or claim that may be made by its manufacturer, is not guaranteed or endorsed by the publisher.
