# Supplementary material for: You’re Prettier When You Smile: Construction and Validation of a Questionnaire to Assess Microaggressions Against Women in the Workplace
Source: Front Psychol. 2022 Mar 16;13:809862. doi: 10.3389/fpsyg.2022.809862 (PMC8966652; doi:10.3389/fpsyg.2022.809862)
Supplement: Supplementary file 1 [file Table_1.DOCX]

| **Table 1.**  *Loadings and Standard Errors of MIMI-16 in Study 1* | | | | | | |
| --- | --- | --- | --- | --- | --- | --- |
| Item | unstandardized loading | | standard error | | standardized loading | |
| Microinvalidations | dataset 1 | dataset 2 | dataset 1 | dataset 2 | dataset 1 | dataset 2 |
| mi1 | 1.000 | 1.000 |  |  | 0.520 | 0.522 |
| mi2 | 2.433 | 2.353 | 0.294 | 0.373 | 0.827 | 0.847 |
| mi3 | 2.116 | 1.844 | 0.267 | 0.296 | 0.739 | 0.712 |
| mi4 | 1.387 | 1.332 | 0.220 | 0.262 | 0.504 | 0.475 |
| mi5 | 1.952 | 1.832 | 0.258 | 0.286 | 0.683 | 0.677 |
| mi6 | 1.492 | 1.326 | 0.212 | 0.262 | 0.603 | 0.562 |
| mi7 | 1.942 | 1.651 | 0.261 | 0.285 | 0.660 | 0.603 |
| mi8 | 2.459 | 2.270 | 0.301 | 0.378 | 0.796 | 0.771 |
| Microinsults |  |  |  |  |  |  |
| mi9 | 1.000 | 1.000 |  |  | 0.594 | 0.718 |
| mi10 | 1.021 | 0.923 | 0.139 | 0.092 | 0.579 | 0.642 |
| mi11 | 0.867 | 0.790 | 0.140 | 0.097 | 0.463 | 0.485 |
| mi12 | 0.450 | 0.456 | 0.084 | 0.103 | 0.406 | 0.453 |
| mi13 | 1.374 | 1.099 | 0.163 | 0.120 | 0.723 | 0.717 |
| mi14 | 0.995 | 0.832 | 0.130 | 0.114 | 0.621 | 0.620 |
| mi15 | 1.048 | 0.839 | 0.134 | 0.103 | 0.645 | 0.629 |
| mi16 | 0.736 | 0.604 | 0.105 | 0.080 | 0.533 | 0.547 |
| *Notes:* MIMI-16 = Microinvalidations and Microinsults Scale, abbreviated items refer to Table 1. | | | | | | |

| **Table 2.**  *Loadings and Standard Errors of MIMI-16 in Study 2* | | | | | | |
| --- | --- | --- | --- | --- | --- | --- |
| Item | unstandardized loading | | SE | | standardized loading | |
| Microinvalidations | dataset 1 | dataset 2 | dataset 1 | dataset 2 | dataset 1 | dataset 2 |
| mi1 | 1.000 | 1.000 |  |  | 0.491 | 0.553 |
| mi2 | 1.684 | 1.807 | 0.213 | 0.169 | 0.786 | 0.856 |
| mi3 | 1.349 | 1.399 | 0.179 | 0.149 | 0.674 | 0.711 |
| mi4 | 1.161 | 1.136 | 0.163 | 0.144 | 0.582 | 0.600 |
| mi5 | 1.560 | 1.469 | 0.184 | 0.158 | 0.713 | 0.713 |
| mi6 | 1.474 | 1.453 | 0.167 | 0.153 | 0.684 | 0.676 |
| mi7 | 1.712 | 1.454 | 0.211 | 0.162 | 0.765 | 0.693 |
| mi8 | 1.732 | 1.616 | 0.216 | 0.152 | 0.809 | 0.778 |
| Microinsults |  |  |  |  |  |  |
| mi9 | 1.000 | 1.000 |  |  | 0.778 | 0.760 |
| mi10 | 0.908 | 0.997 | 0.070 | 0.067 | 0.673 | 0.722 |
| mi11 | 0.654 | 0.726 | 0.076 | 0.072 | 0.513 | 0.539 |
| mi12 | 0.487 | 0.498 | 0.065 | 0.080 | 0.438 | 0.434 |
| mi13 | 1.031 | 0.979 | 0.054 | 0.068 | 0.758 | 0.710 |
| mi14 | 0.638 | 0.859 | 0.068 | 0.076 | 0.528 | 0.621 |
| mi15 | 0.748 | 0.844 | 0.082 | 0.081 | 0.567 | 0.647 |
| mi16 | 0.483 | 0.597 | 0.058 | 0.060 | 0.492 | 0.594 |

*Notes:* MIMI-16 = Microinsults and Microinvalidations Scale, abbreviated items refer to Table 1.
